# Using Voice and Touchscreen Technology to Protect Vulnerable Clients in Residential Care Facilities

**DOI:** 10.1093/geroni/igab046.1258

**Published:** 2021-12-17

**Authors:** Jocelyn Brown, Joan Davitt, Pam Luby, Dorinda Adams

**Affiliations:** 1 University of Maryland Baltimore, Martinsburg, West Virginia, United States; 2 University of Maryland, Baltimore, Maryland, United States; 3 Social Services Administration, Baltimore, Maryland, United States

## Abstract

Due to the COVID-19 pandemic, the Centers for Medicare and Medicaid Services (CMS) issued a memorandum for long term care facilities to restrict visitation by all nonessential personnel, including guardianship case managers. To enhance caseworker access to their guardianship clients, an eastern seaboard state agency distributed Amazon Echo Show devices to clients in long term care facilities. These touchscreen and voice-activated devices have both video and audio capabilities. This study reports the results of the first phase of a comprehensive evaluation, pilot testing the devices via a group of “superuser” case managers to understand the potential challenges and benefits of using these devices. Sixteen case managers participated in two virtual focus groups before and after the installation of an Echo device with one of their guardianship clients. Participants were asked to discuss experiences in accessing clients and client engagement before and after device installation. The focus groups were audio-recorded and transcribed verbatim and two researchers independently identified themes using open and axial coding. Major themes identified included: challenges to device installation and use, strategies to overcome challenges, benefits to using Echo devices, and ethical concerns. These findings suggest that touchscreen or voice-activated devices with video capability can assist case managers in protecting vulnerable clients and ensuring their well-being when in-person access is restricted. Additionally, the devices can be used to enable isolated residents to connect to the outside world, including family, friends, and case managers through technology. Strategies for replication of this innovative program will be discussed.

